# Obesity and Pancreatic Cancer: A Matched-Pair Survival Analysis

**DOI:** 10.3390/jcm9113526

**Published:** 2020-10-31

**Authors:** Patrick Téoule, Erik Rasbach, Hani Oweira, Mirko Otto, Nuh N. Rahbari, Christoph Reissfelder, Felix Rückert, Emrullah Birgin

**Affiliations:** 1 Department of Surgery, Universitätsmedizin Mannheim, Medical Faculty Mannheim, Heidelberg University, Theodor-Kutzer-Ufer 1-3, 68167 Mannheim, Germany; patrick.teoule@umm.de (P.T.); erik.rasbach@umm.de (E.R.); mirko.otto@umm.de (M.O.); nuh.rahbari@umm.de (N.N.R.); christoph.reissfelder@umm.de (C.R.); felix.rueckert@umm.de (F.R.); 2 Hirslanden Hospital Group, Cham, Rigistrasse 1, 6330 Cham, Switzerland; praxis.oweira@hin.ch

**Keywords:** obesity, pancreatic ductal adenocarcinoma, survival, morbidity, mortality

## Abstract

Background: Morbid obesity is a risk factor for pancreatic ductal adenocarcinoma (PDAC). However, the impact of obesity on postoperative outcomes and overall survival in patients with PDAC remains a controversial topic. Methods: Patients who underwent pancreatic surgery for PDAC between 1997 and 2018 were included in this study. Matched pairs (1:1) were generated according to age, gender and American Society of Anesthesiologists status. Obesity was defined according to the WHO definition as BMI ≥ 30 kg/m^2^. The primary endpoint was the difference in overall survival between patients with and without obesity. Results: Out of 553 patients, a total of 76 fully matched pairs were generated. Obese patients had a mean BMI-level of 33 compared to 25 kg/m^2^ in patients without obesity (*p* = 0.001). The frequency of arterial hypertension (*p* = 0.002), intraoperative blood loss (*p* = 0.039), and perineural invasion (*p* = 0.033) were also higher in obese patients. Clinically relevant postoperative complications (*p* = 0.163) and overall survival rates (*p* = 0.885) were comparable in both study groups. Grade II and III obesity resulted in an impaired overall survival, although this was not statistically significant. Subgroup survival analyses revealed no significant differences for completion of adjuvant chemotherapy and curative-intent surgery. Conclusions: Obesity did not affect overall survival and postoperative complications in these patients with PDAC. Therefore, pancreatic surgery should not be withheld from obese patients.

## 1. Introduction

Obesity has become a pandemic affecting more than 400 million people worldwide with the incidence rising [1,2,3,4]. Major complications of obesity are cardiovascular diseases, diabetes mellitus type 2 and the development of several cancers, including pancreatic ductal adenocarcinoma (PDAC) [5,6,7]. PDAC is a devastating malignancy with a rate of overall survival (OS) of about 5%. The aging and growing population is leading to an increase in the rate of pancreatic cancer diagnoses. Surgery and multimodal treatment remain the only curative therapy, but unfortunately, only 20% of patients are candidates for surgery at the time of diagnosis. Despite advances in surgical techniques, perioperative morbidity and mortality rates remain high in pancreatic surgery [8,9]. In obese patients, surgical therapy and perioperative management are even more challenging due to the coexistence of several comorbidities and excessive fat tissue [10]. In the past, several studies have indicated that pancreatectomy for PDAC in obese and overweight patients is associated with high postoperative morbidity and mortality rates [11,12,13,14]. However, the evidence is conflicting as other studies have showed no increased risk of adverse postoperative outcomes in these patients [15,16,17]. Another subject of ongoing debate is the impact of obesity on OS in PDAC. Some studies have reported a poor survival rate in obese patients with PDAC, while others have reported a survival benefit in overweight patients [17,18]. Additionally, the studies reporting on the long-term outcomes regarding a linkage between pancreatic cancer and obesity have employed heterogeneous definitions for obesity although the World Health Organization (WHO) has defined obesity as a body mass index (BMI) level ≥30 kg/m^2^. Furthermore, the impact of obesity on outcomes after pancreatic resections has been analyzed in subgroups including patients with benign lesions [18,19,20,21,22,23,24,25,26]. Given the conflicting data and the global burden of obesity and PDAC, the aim of the present study was to analyze the impact of obesity on postoperative outcome and survival following surgical therapy in patients with PDAC.

## 2. Materials and Methods

### 2.1. Study Design

The ethics committee at the Heidelberg University, Medical Faculty Mannheim, approved this retrospective review of patient charts from a prospectively recorded data base (2015-867R-MA) [27,28]. All consecutive patients who underwent pancreatic surgery for PDAC between 1997 and 2018 at the Department of Surgery, University Hospital Mannheim, Heidelberg University, Medical Faculty Mannheim were screened. Patients with missing clinicopathological and follow-up data were excluded. Matched pairs (1:1) were generated according to the following predefined match criteria for non-modifiable preoperative risk factors associated with OS and obesity: age, gender, and American Society of Anesthesiologists (ASA) status [29,30,31]. Only full matches were accepted. The trial was registered in the German Clinical Trials Register (DRKS00021299). Surgical procedures were performed according to standards as described previously [32,33].

### 2.2. Definitions and Outcomes

Demographic and clinical characteristics included age, sex, BMI, and preoperative status of patients according to the ASA status classification and comorbidities listed for cardiac history (coronary artery disease, congestive heart failure, history of myocardial infarction, artificial valves), pulmonary diseases (chronic obstructive pulmonary disease, pulmonary hypertension, bronchial asthma), arterial hypertension, diabetes mellitus, history of chronic pancreatitis, and preoperative biliary stenting [34]. Histopathological data were analyzed by the Department of Pathology, Universitätsmedizin Mannheim, Mannheim, Germany according to the 6th–8th versions of the TNM classification. The primary endpoint was defined as the OS difference in patients with histologically confirmed PDAC between those with obesity and those without. Secondary endpoints included intraoperative outcome, postoperative morbidity, pathological characteristics, postoperative length of hospital stay, and adjuvant chemotherapy. Obesity was defined according to the WHO criteria as BMI ≥ 30 kg/m^2^ [35,36]. Preoperative laboratory values were assessed as following: albumin (normal: ≥35 g/L), bilirubin (normal: ≤1.2 mg/dL), creatinine (normal: ≤1.4 mg/dL), c-reactive protein (CRP) (normal: ≤2,9 mg/dL), hemoglobin (normal: ≥13 g/dL), platelets (normal: 145–348 × 10^9^/L), and international normalized ratio (INR) (normal: 0.9–1.15). Operative characteristics included operating time (min), total blood loss (mL), surgical details, and perioperative transfusion within 48 h. Postoperative complications were graded in line with the Clavien–Dindo classification if they occurred within 90 days of index operation [37]. In addition, specific complications such as postoperative pancreatic hemorrhage (PPH), postoperative pancreatic fistula (POPF), and postoperative delayed gastric emptying (DGE) were assessed according to the criteria of the International Study Group of Pancreatic Surgery (ISGPS) [38,39,40,41]. Long-term survival was recorded by telephone follow-up in December, 2019, and the survival period or the time of death was noted. Patients lost to follow up were censored.

### 2.3. Statistical Analysis

Categorical data were presented by absolute and relative frequencies (percentage) and compared using Pearson’s χ^2^ test or Fisher’s exact test. Quantitative data were summarized as the mean (standard deviation) or median (interquartile range or 95% confidence interval (95% CI)) and compared depending on the pattern of distribution using the unpaired 2-tailed *t*-test or Wilcoxon test. Survival analysis was done by using the Kaplan–Meier method with the log-rank test. Bonferonni correction was applied for multiple testing. *p*-values < 0.05 were defined as statistically significant. R (version 3.6.1) was used for all statistical analyses.

## 3. Results

### 3.1. Patients Characteristics

**Figure 1:**
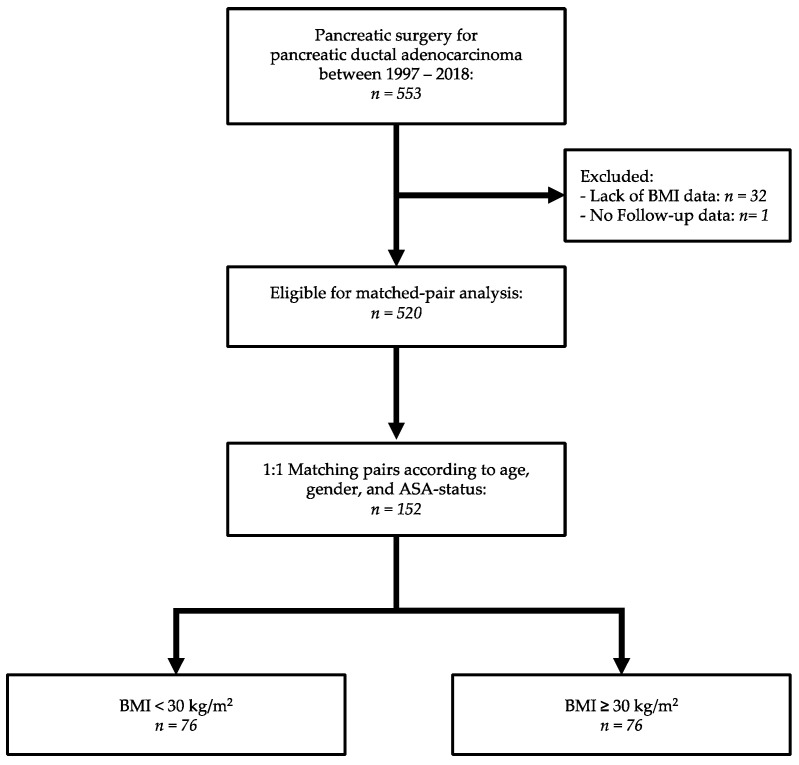
Flow diagram.

Out of 553 patients who underwent surgery for PDAC, a total of 76 fully matched pairs were generated according to the predefined criteria (Figure 1).

Patient characteristics according to the study groups are outlined in Table 1. Obese patients had a mean BMI of 33 kg/m^2^ compared to 25 kg/m^2^ in patients without obesity (*p* = 0.001). The majority of obese patients had grade 1 obesity. There were no patients with ASA status higher than grade III. Patients with obesity had a higher frequency of arterial hypertension than patients without obesity (*n* = 56 (74%) vs. *n* = 36 (47%), *p* = 0.002, respectively). Other baseline characteristics including the values from preoperative laboratory tests were well-balanced between the groups. A total of 46 obese patients (61%) received adjuvant chemotherapy compared to 44 patients (58%) without obesity (*p* = 0.180). As expected, gemcitabine-based chemotherapy was the most commonly employed chemotherapeutic agent. Of note is that five obese patients (*n* = 3 obesity grade 1, *n* = 2 obesity grade 3) discontinued gemcitabine-based chemotherapy due to increased toxicity compared to only one patient in the control group (*p* = 0.102).

### 3.2. Intraoperative Outcome and Pathological Characteristics

The intraoperative and pathological characteristics are summarized in Table 2. Blood loss was higher in obese than in non-obese patients (1050 ± 760 mL vs. 809 ± 607 mL, respectively, *p* = 0.039). Pancreaticoduodenectomies were the most frequently performed procedures in both study groups (*n* = 57 (75%) vs. *n* = 56 (74%), respectively). Six obese patients (8%) had undergone palliative bypass compared to five patients in the control group (7%). Pathological characteristics revealed a higher frequency of perineural invasion in obese than in non-obese patients (*p* = 0.033). No significant differences in tumor size, histologic grading, lymphovascular invasion, and metastasis were observed between obese and non-obese patients.

### 3.3. Postoperative Morbidity

There were no statistically significant differences regarding clinically relevant postoperative complications (Clavien–Dindo ≥III) between the obese and non-obese patients (*n* = 20 (26%) vs. *n* = 12 (16%), respectively, *p* = 0.163) (Table 3). The 30-day mortality rates were 5% in both study groups. Complications such as burst abdomen, radiological or endoscopic interventions, and pancreatic surgery-specific complications (DGE, POPF, and PPH) were equally distributed between the groups. One patient in the non-obese group died of an intraoperative cardiac arrest, whereas the other patients had a multiorgan dysfunction syndrome.

### 3.4. Survival Analyses

**Figure 2:**
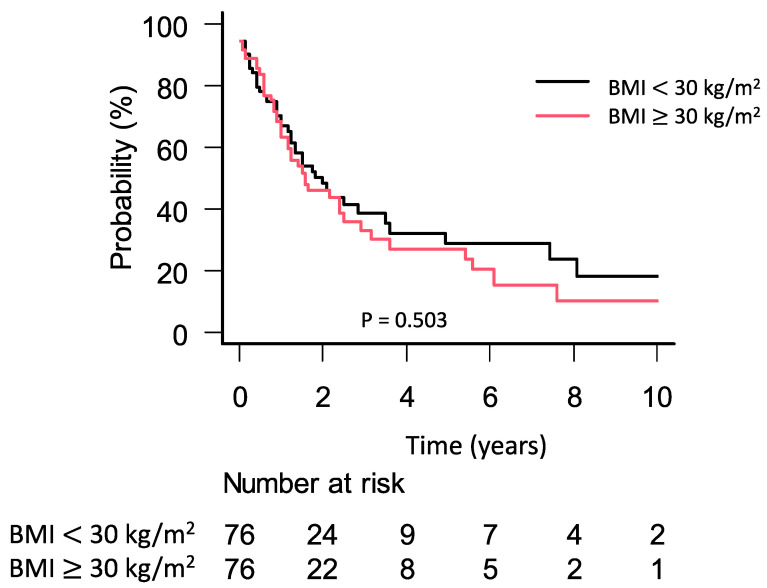
Kaplan–Meier plot for overall survival stratified by BMI ≥ 30 kg/m^2^ and BMI < 30 kg/m^2.^

The median follow-up time was 12 months (interquartile range: 3–26 months). During this period, a total of 84 patients died. Patients with obesity had a median survival of 19 months (95% CI: 12–30 months) compared to 24 months (95% CI: 15–42 months) in patients without obesity (*p* = 0.503; Figure 2). We further stratified the cohort of obese patients according to the definition of obesity grading by the WHO. Patients with grades II and III had an impaired survival outcome compared to the control group but this was not statistically significant (BMI 30–35 kg/m^2^: 26 months (95% CI: 12–38 months); BMI 35–40 kg/m^2^: 16 months (95% CI: 7–16 months); BMI ≥ 40 kg/m^2^: 14 months (0–14 months) *p* = 0.355) (Figure S1, Supplementary Materials). To investigate a potential survival difference in obese patients with surgery of curative intent, we performed further subgroup analysis by excluding patients with palliative bypass surgery (Figure S2). There were no significant differences in OS between the groups (BMI ≥ 30 kg/m^2^: 18 months (95% CI: 12–29 months) vs. BMI < 30 kg/m^2^: 25 months (95% CI: 16–43 months), *p* = 0.239).

Next, we assessed the impact of chemotherapy on OS in patients by excluding patients who did not receive or complete adjuvant or palliative chemotherapy. Six obese patients discontinued chemotherapy due to increased toxicity (*n* = 1 FOLFIRNOX, *n* = 5 gemcitabine-based chemotherapy), compared to one patient in the control group (*p* = 0.056). We detected a lower median survival rate in obese patients than in the control group (19 months (95% CI: 11–43 months) vs. 42 months (95% CI: 21–84 months, respectively)), however, this did not reach statistical significance (*p* = 0.133) (Figure S3).

## 4. Discussion

Surgery and adjuvant chemotherapy constitute the standard of care for curative therapy for PDAC [42]. In the last three decades, advances in oncological treatment strategies have markedly improved OS in PDAC. Still, there is a lack of data regarding predictors for long-term survival and risk stratification of patients suffering from PDAC as the survival rates are highly variable in certain patient populations [43,44,45,46,47]. Obesity represents a major risk factor for developing pancreatic cancer [6,48]. Due to a rising incidence of obesity and PDAC, the surgical treatment of obese patients with pancreatic cancer is expected to increase further [4,49]. However, inconsistent postoperative outcomes and oncological results have been outlined in previous studies that included obese patients with PDAC following oncological therapy. This was mainly due to the use of heterogeneous definitions of obesity and inhomogeneous patient cohorts as well as variable surgical techniques [11,12,13,14,15,16,17,18,19,20,21,22,23,24,25,26,50]. Some authors detected comparable OS rates in obese and non-obese patients (19.8 months vs. 23.5 months, respectively; *p* = 0.46) [11], while others observed a worse survival in patients with BMI > 35 kg/m^2^ than in those with BMI < 23 kg/m^2^ (13.2 months vs. 27.4 months, respectively; *p* = 0.02) [18]. Tsai et al. even reported improved OS in patients with a BMI > 25 than in patients with a normal weight following pancreaticoduodenectomy (20.3 and 20.1 months vs. 14.6 months, respectively) [17]. Therefore, we addressed this lack of evidence by analyzing a fully matched pair analysis of 152 patients with PDAC and surgical treatment in a tertiary referral center. We matched the groups according to age, gender, ASA status, and BMI-level and detected a comparable long-term survival in patients with BMI ≥ 30 kg/m^2^ and in non-obese patients.

Age, gender, chronic pancreatitis, and modifiable factors such as obesity and metabolic syndrome-related diabetes are well-known risk factors for PDAC. Chronic pancreatitis and obesity are associated with desmoplasia in pancreatic cancer. However, the effect of desmoplasia is not fully understood and remains a matter of controversy [51,52]. Desmoplasia represents a two-edged sword. On the one hand, it has been reported to prevent local tumor invasion through encapsulation of the tumor by fibrous tissue, but on the other hand, desmoplasia might cause a poor response to chemotherapy due to reduced tumor vascularization resulting in defective drug delivery to the tumor [51,52]. In a previous study, we showed that desmoplastic reaction in patients with chronic pancreatitis and pancreatic cancer had no impact on survival after resection with curative intent [27]. As obese patients are accompanied by severe comorbidities, adjuvant therapy for them can be more challenging and result in dose reductions or even discontinuation of therapy [53]. In the present study, although the risk factors posed by diabetes and the history of chronic pancreatitis were not matched, we detected no difference among the distribution in these groups. Moreover, adjuvant therapy was well balanced between the groups, although a higher proportion of obese patients discontinued chemotherapy following surgery. This was reflected by the slightly worse survival rate in obese patients detected in the subgroup analysis of patients who received adjuvant chemotherapy although this did not reach statistical significance.

Obesity is characterized by changes in cancer-associated adipokine production [54]. Moreover, DNA damage pathways, adipokines, and proinflammatory environment related to obesity contribute to angiogenesis and metastasis with advanced tumor size, residual tumor and nodal involvement [17,18]. In line with these findings, obese patients exhibited a higher frequency of perineural invasion in the present study, indicating more aggressive tumor biology. We also observed a higher frequency of nodal involvement in obese patients although this did not reach statistical significance.

Furthermore, obesity is known to be associated with an increased risk of cardiovascular diseases, diabetes, and premature death [55]. Previous studies have found that surgical therapy of obese patients with PDAC is associated with high morbidity rates (e.g., intraabdominal abscess- and/or fluid collections, wound infection rates, and POPF) of up to 68% [11,12,13,14]. The present study demonstrates that obesity is not associated with increased postoperative overall complications, pancreatic surgery-specific complications, length of stay, and 90-day mortality rates as described in the literature [16,56,57,58].

Moreover, while the operating time was comparable for both study groups, intraoperative blood loss was significantly increased in obese patients, reflecting a more demanding resection in obese patients, which is in line with other studies [16,17]. Still, one has to consider the intraoperative difficulties arising in obese patients from a higher amount of visceral fat tissue, which demand special surgical expertise and equipment (e.g., retractors, positioning). Therefore, oncological treatment of obese patients with PDAC should be restricted to high-volume pancreatic centers with experience in handling obese patients [59,60].

There are some limitations to the present study. First, this is a single-center study with a retrospective data study design. Therefore, there will be some selection and reporting bias. Second, there are some missing values for pathological characteristics in this prospectively recorded data base, which is mainly due to the non-standardized assessment of perineural or lymphatic invasion before 2009. Third, the patient cohort consists of mainly patients with grade I obesity. Although we detected impaired survival in patients with grade II and III obesity, this was not statistically significant. This was probably due to the small sample size of patients with high-grade obesity. Fourth, the follow-up time was rather short in the present analysis. This was mainly due to the number of censored patients and the high number of patients who died during the follow-up period (up to 55%).

## 5. Conclusions

Within the above-mentioned limitations, obesity was not associated with worse postoperative outcomes and long-term survival despite a predominance of perineural invasion and higher intraoperative blood loss. However, the impact of high-grade obesity still remains unclear.

These findings should therefore be considered when assessing patients for operation and when counseling patients about their operative risk. Nevertheless, pancreatic surgery should not be withheld for obese PDAC patients when it is oncologically appropriate. There is urgent need for multicenter- or register-based studies to validate the present results before definitive conclusions can be drawn.

## Figures and Tables

**Table 1:** Characteristics of the study groups.

Variables	BMI < 30 kg/m^2^ *n* = 76 (%)	BMI ≥ 30 kg/m^2^ *n* = 76 (%)	*p*-Value
Age, years *	68 (9)	68 (9)	≥0.99
Sex ratio (M:F)	38:38	38:38	≥0.99
BMI *	25 (3)	33 (4)	0.001
BMI 30–35 kg/m^2^	-	64 (84)	
BMI 35–40 kg/m^2^	-	8 (10)	
BMI **≤** 40 kg/m^2^	-	4 (6)	
ASA			≥0.99
I	25 (33)	25 (33)	
II	18 (24)	18 (24)	
III	33 (43)	33 (43)	
Cardiac comorbidity	29 (38)	25 (33)	0.611
Pulmonary comorbidity	10 (13)	17 (22)	0.202
Arterial hypertension	36 (47)	56 (74)	0.002
Diabetes mellitus	28 (37)	37 (49)	0.189
History of chronic pancreatitis	9 (12)	10 (13)	≥0.99
Preoperative stenting	4 (6)	4 (6)	≥0.99
Preoperative blood values *			
Albumin (g/L)	26 (14)	27 (13)	0.776
Bilirubin (mg/dL)	5.3 (5.9)	5.6 (6.2)	0.758
Creatinine (mg/dL)	0.9 (0.3)	0.9 (0.2)	0.313
C-reactive protein (mg/dL)	16 (23)	22 (40)	0.304
Hemoglobin (g/dL)	12.8 (1.6)	12.7 (1.4)	0.583
Platelets (10E9/L)	275 (76)	255 (107)	0.407
International normalized ratio	1.0 (0.1)	1.0 (0.1)	0.869
Adjuvant Chemotherapy	44 (58)	46 (61)	0.330
Chemotherapeutic regimen			0.232
Single agent	37 (49)	36 (47)	
Double agents	5 (7)	2 (3)	
Triple agents	1 (1)	4 (5)	
X	1 (1)	4 (5)	
Additional targeted therapy	4 (5)	2 (3)	0.431
Radiation Therapy	1 (1)	1 (1)	0.360
Radiochemotherapy	3 (4)	1 (1)	≥0.99

ASA: American Society of Anesthesiologists; BMI: body mass index; X: missing data; *: values are mean (s.d.).

**Table 2:** Intraoperative and pathological characteristics.

Variables	BMI < 30 kg/m^2^ *n* = 76 (%)	BMI ≥ 30 kg/m^2^ *n* = 76 (%)	*p*-Value
Operating time (min) *	333 (100)	360 (104)	0.108
Blood loss (mL) *	809 (607)	1050 (760)	0.039
Type of surgery			≥0.99
Whipple	8 (11)	9 (12)	
PPPD	48 (63)	48 (63)	
Total pancreatectomy	4 (5)	6 (8)	
Distal pancreatectomy	8 (11)	8 (11)	
Palliative Bypass	6 (8)	5 (7)	
Vascular resection	38 (50)	37 (52)	≥0.99
Perioperative blood transfusion	17 (22)	23 (30)	0.357
T classification			0.705
1	1 (1)	3 (4)	
2	6 (8)	9 (12)	
3	59 (78)	57 (75)	
4	5 (7)	4 (5)	
X	5 (7)	2 (3)	
Nodal status			0.150
0	24 (32)	22 (29)	
1	44 (58)	49 (64)	
2	2 (3)	0 (0)	
X	6 (8)	5 (7)	
M status			0.882
0	71 (93)	71 (93)	
1	4 (5)	5 (7)	
X	1 (1)	0 (0)	
Grading			0.748
1	1 (1)	2 (3)	
2	41 (54)	37 (49)	
3	25 (33)	31 (41)	
4	1 (1)	0 (0)	
X	8 (11)	6 (8)	
Resection status			0.344
0	51 (67)	59 (78)	
1	15 (20)	9 (12)	
2	1 (1)	3 (4)	
X	10 (13)	5 (7)	
Perineural invasion			0.033
0	21 (28)	9 (12)	
1	32 (42)	40 (53)	
x	23 (30)	27 (36)	
Lymphatic invasion			0.570
0	22 (29)	17 (22)	
1	32 (42)	38 (50)	
X	22 (29)	21(28)	
Vascular invasion			≥0.99
0	39 (51)	40 (53)	
1	30 (39)	31(41)	
X	6 (8)	5 (7)	

BMI: body mass index; PPPD: Pylorus-preserving pancreatoduodenectomy; X: missing data; *: values are mean (s.d.).

**Table 3:** Postoperative outcome.

Variables	BMI < 30 kg/m^2^ *n* = 76 (%)	BMI ≥ 30 kg/m^2^ *n* = 76 (%)	*p*-Value
Clavien–Dindo Classification			0.645
Grade I	14 (18)	10 (13)	
Grade II	15 (20)	12 (16)	
Grade III	6 (8)	13 (17)	
Grade IV	2 (3)	2 (3)	
Grade V (death)	4 (5)	5 (7)	
30-day mortality rate	4 (5)	4 (5)	≥0.99
Clinically-relevant complications	12 (16)	20 (26)	0.163
Surgical site infection	7 (9)	5 (7)	0.765
Burst abdomen	1 (1)	2 (3)	0.559
Invasive interventions	4 (5)	8 (11)	0.368
Endoscopic intervention	2 (3)	5 (7)	0.442
Radiologic intervention	2 (3)	4 (5)	0.681
DGE			0.558
Grade B/C	5 (7)	8 (11)	
POPF			0.582
Grade B/C	6 (8)	8 (11)	
PPH			0.360
Grade B/C	3 (4)	1 (1)	
Length of hospital stay (d) *	15 (13–20)	16 (13–25)	0.201

BMI: body mass index; DGE: delayed gastric emptying; POPF: postoperative pancreatic fistula; PPH: postoperative pancreatic hemorrhage; *: Values are the median (iqr).

## Supplementary Materials

The following are available online at https://www.mdpi.com/2077-0383/9/11/3526/s1, Figure S1: Kaplan–Meier plot for overall survival stratified by WHO grading of obesity, Figure S2: Kaplan–Meier plot for overall survival in patients with curative-intent surgery, Figure S3: Kaplan–Meier plot for overall survival in patients with completed adjuvant chemotherapy.

## References

[1] Popkin B.M. (2006). Global nutrition dynamics: The world is shifting rapidly toward a diet linked with noncommunicable diseases. Am. J. Clin. Nutr..

[2] Popkin B.M., Slining M.M. (2013). New dynamics in global obesity facing low- and middle-income countries. Obes. Rev..

[3] Wang G.R., Li L., Pan Y.H., Tian G.D., Lin W.L., Li Z., Chen Z.Y., Gong Y.L., Kikano G.E., Stange K.C. (2013). Prevalence of metabolic syndrome among urban community residents in China. BMC Pub. Health.

[4] (2006). Centers for Disease Control and Prevention (CDC) State-specific prevalence of obesity among adults-United States, 2005. MMWR Morb. Mortal. Wkly. Rep..

[5] Berger N.A. (2014). Obesity and cancer pathogenesis. Ann. N. Y. Acad. Sci..

[6] Renehan A.G., Tyson M., Egger M., Heller R.F., Zwahlen M. (2008). Body-mass index and incidence of cancer: A systematic review and meta-analysis of prospective observational studies. Lancet.

[7] Lauby-Secretan B., Scoccianti C., Loomis D., Grosse Y., Bianchini F., Straif K. (2016). International Agency for Research on Cancer Handbook Working Group Body Fatness and Cancer--Viewpoint of the IARC Working Group. N. Engl. J. Med..

[8] Téoule P., Bartel F., Birgin E., Rückert F., Wilhelm T.J. (2019). The Clavien-Dindo Classification in Pancreatic Surgery: A Clinical and Economic Validation. J. Invest. Surg..

[9] Nimptsch U., Krautz C., Weber G.F., Mansky T., Grützmann R. (2016). Nationwide In-hospital Mortality Following Pancreatic Surgery in Germany is Higher than Anticipated. Ann. Surg..

[10] Adams K.F., Schatzkin A., Harris T.B., Kipnis V., Mouw T., Ballard-Barbash R., Hollenbeck A., Leitzmann M.F. (2006). Overweight, Obesity, and Mortality in a Large Prospective Cohort of Persons 50 to 71 Years Old. N. Engl. J. Med..

[11] Benns M., Woodall C., Scoggins C., McMasters K., Martin R. (2009). The impact of obesity on outcomes following pancreatectomy for malignancy. Ann. Surg. Oncol..

[12] Williams T.K., Rosato E.L., Kennedy E.P., Chojnacki K.A., Andrel J., Hyslop T., Doria C., Sauter P.K., Bloom J., Yeo C.J. (2009). Impact of obesity on perioperative morbidity and mortality after pancreaticoduodenectomy. J. Am. Coll. Surg..

[13] Su Z., Koga R., Saiura A., Natori T., Yamaguchi T., Yamamoto J. (2010). Factors influencing infectious complications after pancreatoduodenectomy. J. Hepatobil. Pancreat. Sci.

[14] House M.G., Fong Y., Arnaoutakis D.J., Sharma R., Winston C.B., Protic M., Gonen M., Olson S.H., Kurtz R.C., Brennan M.F. (2008). Preoperative predictors for complications after pancreaticoduodenectomy: Impact of BMI and body fat distribution. J. Gastrointest. Surg..

[15] Balentine C.J., Enriquez J., Cruz G., Hodges S., Bansal V., Jo E., Ahern C., Sansgiry S., Petersen N., Silberfein E. (2011). Obesity does not increase complications following pancreatic surgery. J. Surg. Res..

[16] Del Chiaro M., Rangelova E., Ansorge C., Blomberg J., Segersvärd R. (2013). Impact of body mass index for patients undergoing pancreaticoduodenectomy. World J. Gastroint. Pathophysiol..

[17] Tsai S., Choti M.A., Assumpcao L., Cameron J.L., Gleisner A.L., Herman J.M., Eckhauser F., Edil B.H., Schulick R.D., Wolfgang C.L. (2010). Impact of obesity on perioperative outcomes and survival following pancreaticoduodenectomy for pancreatic cancer: A large single-institution study. J. Gastrointest. Surg..

[18] Fleming J.B., Gonzalez R.J., Petzel M.Q.B., Lin E., Morris J.S., Gomez H., Lee J.E., Crane C.H., Pisters P.W.T., Evans D.B. (2009). Influence of obesity on cancer-related outcomes after pancreatectomy to treat pancreatic adenocarcinoma. Arch. Surg..

[19] Gaujoux S., Cortes A., Couvelard A., Noullet S., Clavel L., Rebours V., Lévy P., Sauvanet A., Ruszniewski P., Belghiti J. (2010). Fatty pancreas and increased body mass index are risk factors of pancreatic fistula after pancreaticoduodenectomy. Surgery.

[20] Li D., Morris J.S., Liu J., Hassan M.M., Day R.S., Bondy M.L., Abbruzzese J.L. (2009). Body Mass Index and Risk, Age of Onset, and Survival in Patients With Pancreatic Cancer. JAMA.

[21] McWilliams R.R., Matsumoto M.E., Burch P.A., Kim G.P., Halfdanarson T.R., de Andrade M., Reid-Lombardo K., Bamlet W.R. (2010). Obesity Adversely Affects Survival in Pancreatic Cancer Patients. Cancer.

[22] Yuan C., Bao Y., Wu C., Kraft P., Ogino S., Ng K., Qian Z.R., Rubinson D.A., Stampfer M.J., Giovannucci E.L. (2013). Prediagnostic Body Mass Index and Pancreatic Cancer Survival. J. Clin. Oncol..

[23] Kasenda B., Bass A., Koeberle D., Pestalozzi B., Borner M., Herrmann R., Jost L., Lohri A., Hess V. (2014). Survival in overweight patients with advanced pancreatic carcinoma: A multicentre cohort study. BMC Cancer.

[24] Olson S.H., Chou J.F., Ludwig E., O’Reilly E., Allen P.J., Jarnagin W.R., Bayuga S., Simon J., Gonen M., Reisacher W.R. (2010). Allergies, obesity, other risk factors and survival from pancreatic cancer. Int. J. Cancer.

[25] Pelucchi C., Galeone C., Polesel J., Manzari M., Zucchetto A., Talamini R., Franceschi S., Negri E., La Vecchia C. (2014). Smoking and body mass index and survival in pancreatic cancer patients. Pancreas.

[26] Dandona M., Linehan D., Hawkins W., Strasberg S., Gao F., Wang-Gillam A. (2011). Influence of obesity and other risk factors on survival outcomes in patients undergoing pancreaticoduodenectomy for pancreatic cancer. Pancreas.

[27] Birgin E., Hablawetz P., Téoule P., Rückert F., Wilhelm T.J. (2018). Chronic pancreatitis and resectable synchronous pancreatic carcinoma: A survival analysis. Pancreatology.

[28] Birgin E., Reeg A., Téoule P., Rahbari N.N., Post S., Reissfelder C., Rückert F. (2019). Early postoperative pancreatitis following pancreaticoduodenectomy: What is clinically relevant postoperative pancreatitis?. HPB (Oxford).

[29] Hartwig W., Gluth A., Hinz U., Koliogiannis D., Strobel O., Hackert T., Werner J., Büchler M.W. (2016). Outcomes after extended pancreatectomy in patients with borderline resectable and locally advanced pancreatic cancer. Br. J. Surg..

[30] Albrecht R., Haase D., Zippel R., Koch H., Settmacher U. (2017). Robot-assisted surgery—Progress or expensive toy?: Matched-pair comparative analysis of robot-assisted cholecystectomy vs. laparoscopic cholecystectomy. Chirurg.

[31] Deichmann S., Bolm L.R., Honselmann K.C., Wellner U.F., Lapshyn H., Keck T., Bausch D. (2018). Perioperative and Long-term Oncological Results of Minimally Invasive Pancreatoduodenectomy as Hybrid Technique—A Matched Pair Analysis of 120 Cases. Zentralbl. Chir..

[32] Téoule P., Kunz B., Schwarzbach M., Birgin E., Rückert F., Wilhelm T.J., Niedergethmann M., Post S., Rahbari N.N., Reißfelder C. (2019). Influence of Clinical pathways on treatment and outcome quality for patients undergoing pancreatoduodenectomy?—A retrospective outcome cohort study. Asian. J. Surg..

[33] Téoule P., Römling L., Schwarzbach M., Birgin E., Rückert F., Wilhelm T.J., Niedergethmann M., Post S., Rahbari N.N., Reißfelder C. (2019). Clinical Pathways For Pancreatic Surgery: Are They A Suitable Instrument For Process Standardization To Improve Process And Outcome Quality Of Patients Undergoing Distal And Total Pancreatectomy?—A Retrospective Cohort Study. Ther. Clin. Risk. Manag..

[34] Saklad M. (1941). Grading of Patients for Surgical Procedures. Anesthesiology.

[35] (2004). WHO Expert Consultation Appropriate body-mass index for Asian populations and its implications for policy and intervention strategies. Lancet.

[36] (2000). Obesity: Preventing and managing the global epidemic. Report of a WHO consultation. World Health Organ. Tech. Rep. Ser..

[37] Dindo D., Demartines N., Clavien P.A. (2004). Classification of surgical complications: A new proposal with evaluation in a cohort of 6336 patients and results of a survey. Ann. Surg..

[38] Bassi C., Marchegiani G., Dervenis C., Sarr M., Abu Hilal M., Adham M., Allen P., Andersson R., Asbun H.J., Besselink M.G. (2017). The 2016 update of the International Study Group (ISGPS) definition and grading of postoperative pancreatic fistula: 11 Years After. Surgery.

[39] Wente M.N., Bassi C., Dervenis C., Fingerhut A., Gouma D.J., Izbicki J.R., Neoptolemos J.P., Padbury R.T., Sarr M.G., Traverso L.W. (2007). Delayed gastric emptying (DGE) after pancreatic surgery: A suggested definition by the International Study Group of Pancreatic Surgery (ISGPS). Surgery.

[40] Bassi C., Dervenis C., Butturini G., Fingerhut A., Yeo C., Izbicki J., Neoptolemos J., Sarr M., Traverso W., Buchler M. (2005). Postoperative pancreatic fistula: An international study group (ISGPF) definition. Surgery.

[41] Wente M.N., Veit J.A., Bassi C., Dervenis C., Fingerhut A., Gouma D.J., Izbicki J.R., Neoptolemos J.P., Padbury R.T., Sarr M.G. (2007). Postpancreatectomy hemorrhage (PPH): An International Study Group of Pancreatic Surgery (ISGPS) definition. Surgery.

[42] Malvezzi M., Bertuccio P., Levi F., La Vecchia C., Negri E. (2014). European cancer mortality predictions for the year 2014. Ann. Oncol..

[43] Lim J.E., Chien M.W., Earle C.C. (2003). Prognostic factors following curative resection for pancreatic adenocarcinoma: A population-based, linked database analysis of 396 patients. Ann. Surg..

[44] Sohn T.A., Yeo C.J., Cameron J.L., Koniaris L., Kaushal S., Abrams R.A., Sauter P.K., Coleman J., Hruban R.H., Lillemoe K.D. (2000). Resected adenocarcinoma of the pancreas-616 patients: Results, outcomes, and prognostic indicators. J. Gastrointest. Surg..

[45] Pawlik T.M., Gleisner A.L., Cameron J.L., Winter J.M., Assumpcao L., Lillemoe K.D., Wolfgang C., Hruban R.H., Schulick R.D., Yeo C.J. (2007). Prognostic relevance of lymph node ratio following pancreaticoduodenectomy for pancreatic cancer. Surgery.

[46] Riediger H., Keck T., Wellner U., zur Hausen A., Adam U., Hopt U.T., Makowiec F. (2009). The lymph node ratio is the strongest prognostic factor after resection of pancreatic cancer. J. Gastrointest. Surg..

[47] Stark A.P., Sacks G.D., Rochefort M.M., Donahue T.R., Reber H.A., Tomlinson J.S., Dawson D.W., Eibl G., Hines O.J. (2016). Long-term Survival in Patients with Pancreatic Ductal Adenocarcinoma. Surgery.

[48] Aune D., Greenwood D.C., Chan D.S.M., Vieira R., Vieira A.R., Navarro Rosenblatt D.A., Cade J.E., Burley V.J., Norat T. (2012). Body mass index, abdominal fatness and pancreatic cancer risk: A systematic review and non-linear dose-response meta-analysis of prospective studies. Ann. Oncol..

[49] Siegel R.L., Miller K.D., Jemal A. (2018). Cancer statistics, 2018. CA Cancer J. Clin..

[50] Dindo D., Muller M.K., Weber M., Clavien P.-A. (2003). Obesity in general elective surgery. Lancet.

[51] Lunevicius R., Nakanishi H., Ito S., Kozaki K., Kato T., Tatematsu M., Yasui K. (2001). Clinicopathological significance of fibrotic capsule formation around liver metastasis from colorectal cancer. J. Cancer Res. Clin. Oncol..

[52] Incio J., Liu H., Suboj P., Chin S.M., Chen I.X., Pinter M., Ng M.R., Nia H.T., Grahovac J., Kao S. (2016). Obesity-induced inflammation and desmoplasia promote pancreatic cancer progression and resistance to chemotherapy. Cancer Discov..

[53] Renehan A.G., Harvie M., Cutress R.I., Leitzmann M., Pischon T., Howell S., Howell A. (2016). How to Manage the Obese Patient With Cancer. JCO.

[54] Chang H.-H., Eibl G. (2019). Obesity-Induced Adipose Tissue Inflammation as a Strong Promotional Factor for Pancreatic Ductal Adenocarcinoma. Cells.

[55] Van Gaal L.F., Mertens I.L., De Block C.E. (2006). Mechanisms linking obesity with cardiovascular disease. Nature.

[56] Nazzani S., Preisser F., Mazzone E., Tian Z., Mistretta F.A., Shariat S.F., Saad F., Graefen M., Tilki D., Montanari E. (2018). In-hospital length of stay after major surgical oncological procedures. Eur. J. Surg. Oncol..

[57] Müller M.W., Friess H., Kleeff J., Dahmen R., Wagner M., Hinz U., Breisch-Girbig D., Ceyhan G.O., Büchler M.W. (2007). Is there still a role for total pancreatectomy?. Ann. Surg..

[58] Fisher W.E., Hodges S.E., Wu M.-F., Hilsenbeck S.G., Brunicardi F.C. (2012). Assessment of the learning curve for pancreaticoduodenectomy. Am. J. Surg..

[59] Richter A., Niedergethmann M., Sturm J.W., Lorenz D., Post S., Trede M. (2003). Long-term results of partial pancreaticoduodenectomy for ductal adenocarcinoma of the pancreatic head: 25-year experience. World J. Surg..

[60] Karampinis I., Lion E., Hetjens S., Vassilev G., Galata C., Reissfelder C., Otto M. (2020). Trocar Site HERnias after Bariatric Laparoscopic Surgery (HERBALS): A Prospective Cohort Study. Obes. Surg..

