# Supplementary material for: Obesity and Pancreatic Cancer: A Matched-Pair Survival Analysis
Source: J Clin Med. 2020 Oct 31;9(11):3526. doi: 10.3390/jcm9113526 (PMC7693315; doi:10.3390/jcm9113526)

Figure S1 Kaplan-Meier plot for overall survival stratified by WHO grading of obesity

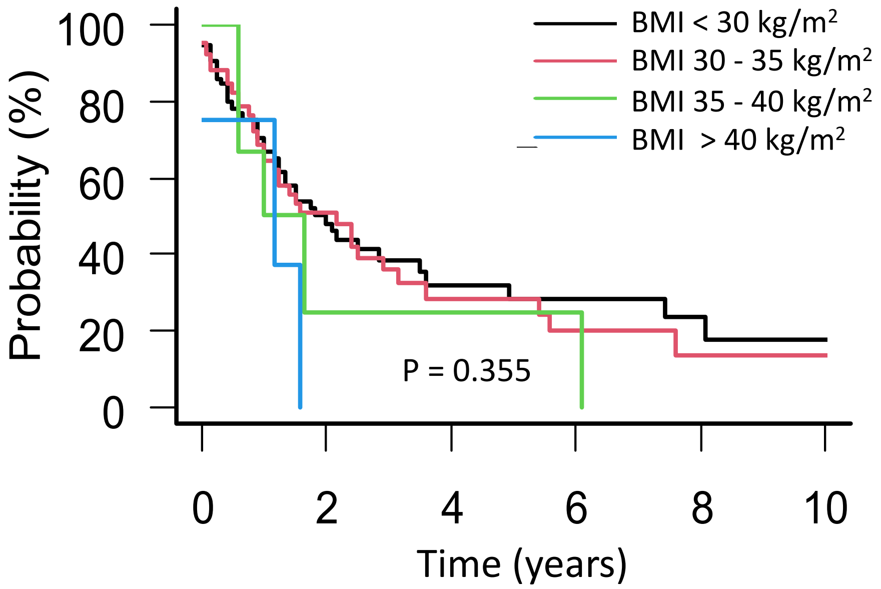

Number at risk

BMI < 30 kg/m²	76	24	9	7	4	2
BMI 30 - 35 kg/m²	64	21	7	4	2	1
BMI 35 - 40 kg/m²	8	1	1	1	0	0
BMI > 40 kg/m²	4	0	0	0	0	0

Figure S2 Kaplan-Meier plot for overall survival in patients with curative-intent surgery

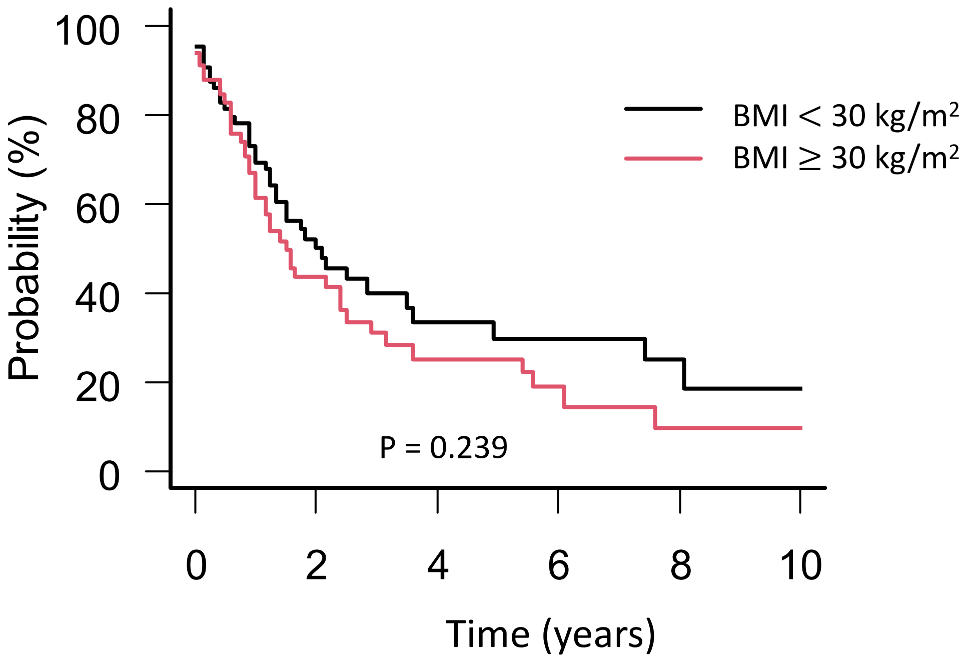

Number at risk

BMI < 30 kg/m^2	70	24	9	7	4	2
BMI ≥ 30 kg/m^2	71	21	8	5	2	1

Figure S3 Kaplan-Meier plot for overall survival in patients with completed adjuvant chemotherapy

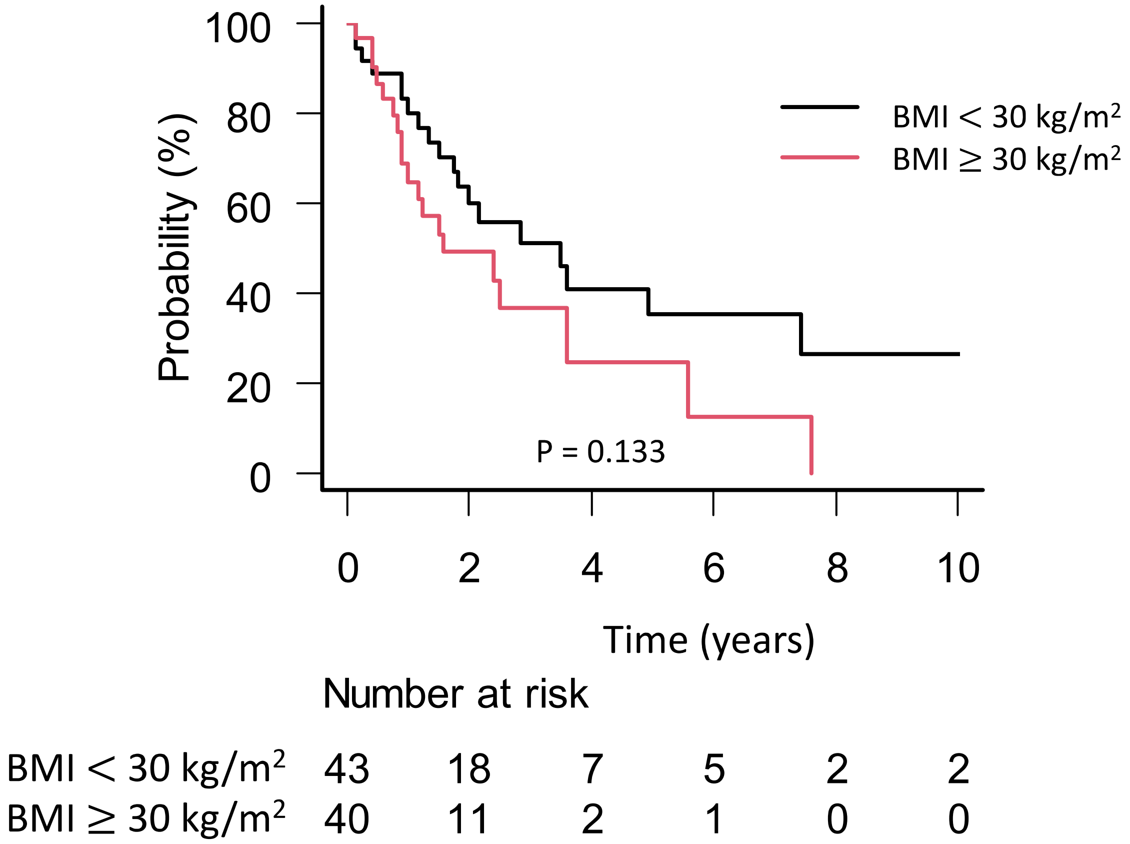

Supplement: Supplementary file 1 [file jcm-09-03526-s001.pdf]
